# A biobanking turning‐point in the use of formalin‐fixed, paraffin tumor blocks to unveil kinase signaling in melanoma

**DOI:** 10.1002/ctm2.466

**Published:** 2021-08-04

**Authors:** Erika Velasquez, Leticia Szadai, Qimin Zhou, Yonghyo Kim, Indira Pla, Aniel Sanchez, Roger Appelqvist, Henriett Oskolas, Matilda Marko‐Varga, Boram Lee, Ho Jeong Kwon, Johan Malm, Attila Marcell Szász, Jeovanis Gil, Lazaro Hiram Betancourt, István Balázs Németh, György Marko‐Varga

**Affiliations:** ^1^ Section for Clinical Chemistry, Department of Translational Medicine, Lund University Skåne University Hospital Malmö Malmö Sweden; ^2^ Division of Oncology and Pathology, Department of Clinical Sciences Lund Lund University Lund Sweden; ^3^ Treat4Life AB Malmö Sweden; ^4^ Chemical Genomics Global Research Lab, Department of Biotechnology, College of Life Science and Biotechnology Yonsei University Seoul Republic of Korea; ^5^ Department of Bioinformatics Semmelweis University Budapest Hungary; ^6^ Department of Dermatology and Allergology University of Szeged Szeged Hungar; ^7^ 1st Department of Surgery Tokyo Medical University Tokyo Japan; ^8^ Clinical Protein Science and Imaging, Biomedical Centre, Department of Biomedical Engineering Lund University Lund Sweden; ^9^ Department of Plastic and Reconstructive Surgery, Shanghai Ninth People's Hospital Shanghai Jiao Tong University School of Medicine Shanghai China

**Abbreviations**
BRAF: serine/threonine‐protein kinase B‐raf
ERK: extracellular signal‐regulated kinase
FFPE: formalin‐fixed and paraffin‐embedded
FFT: fresh frozen tumors
MEK: mitogen‐activated protein kinase
MM: malignant melanoma
MS: mass spectrometry
PTMs: posttranslational modifications

Dear Editor,

Malignant melanoma (MM) is one of the most aggressive human solid tumors, and it is associated with the highest mortality of all skin cancers.^1^ Aberrant patterns of protein expression and posttranslational modifications (PTMs) have been linked to MM pathogenesis,^2^ which has promoted large‐scale proteomics studies. Our study establishes a novel proteomics strategy that provides validity for formalin‐fixed and paraffin‐embedded (FFPE) tumors as a novel source for phosphoprotein mapping in MM. This constitutes an important future clinical resource for discovering novel biomarkers and refining therapeutic approaches for MM treatment.

Most of the MM proteomic analyses using patient samples to date have been conducted on fresh frozen tumors (FFT). However, FFT specimens are difficult to preserve with a limited number of samples in biobanks compared to FFPE tissues.^3^ Roughly 500 million FFPE cancer tissues are stored in biobank archives linked to relevant clinical information.^4^ This represents a precious resource for the elucidation of novel molecular mechanisms and new biomarkers for MM, as well as a potential tool for personalized treatment monitoring. So far, a comparison between the proteomics profiles of FFPE tumor blocks and FFT in the context of MM has not been addressed. Only a few studies have focused on FFPE samples to explore the MM proteome, but none of these has evaluated the preservation of PTMs, such as phosphorylation.^5^ This is important since alterations in the phosphorylation status of the tumor are linked to the activation of oncogenic pathways.^6^

Here, we analyzed tumor samples derived from 11 patients diagnosed with MM. After surgical removal, all tumors were cut into several pieces (*n* = 49). For nine patients, tumor pieces were preserved in a paired fashion, two as FFT and one as FFPE. Three tumor layers of FFPE blocks were analyzed. For two of the patients, we only had tissues preserved with one of the methods, FFT or FFPE. In total, six primary tumors (*n* = 24 samples) and five tumor metastases (*n* = 25 samples) were analyzed. The histopathological characterization of the samples was performed (Table S1). Critical parameters such as tumor content, percentage of stroma, degree of necrosis, and lymphocyte infiltration were also considered. Samples were subjected to a streamlined proteomics‐phosphoproteomics and bioinformatics pipeline^7^ (Figure S1 and Supporting File 1).

Global proteome analysis resulted in the identification and quantitation of 7624 protein groups across all data sets (Figures 1A–1D, Table S2). Figure 1A shows the total number of proteins identified per patient and preservation conditions. FFT versus FFPE analysis displayed comparable proteome coverages, with an overlap of ∼99.8% and ∼98.3% for protein and peptide identifications, respectively (Figures 1A and 1B). A strong quantitative correlation between the two conditions was also observed (Pearson correlation, *r* = 0.97) (Figure 1C, Figure S2). The 2D functional annotation enrichment analysis^8^ unveils similar biological pathways between primary tumors and metastases regardless of storage condition (Figure 1D). Primary tumors were enriched in pathways linked to the epidermis development and differentiation, and extracellular matrix organization. In contrast, metastases displayed pathways related to cell proliferation, RNA metabolism and transcription.

**FIGURE 1:**
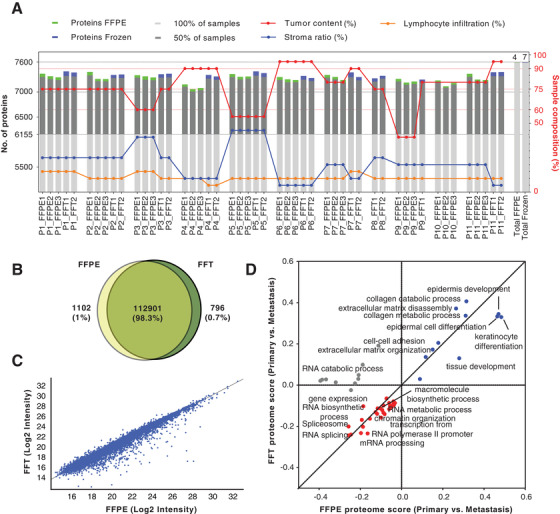
Global proteome analysis of FFPE and FFT tumor samples. (A) Bar graphs showing the proteome coverage by patient and condition, and its association with histological parameters. (B) Venn diagram comparing the overlap between FFPE and FFT samples at the level of peptide identifications and showing the total number of peptides identified across all patients in each condition. (C) Scatter plot of the correlation between the median values of the protein intensities of all identifications in FFT and FFPE samples, across all patients (Pearson correlation, *r* = 0.97). (D) 2‐D pathway annotation enrichment analysis performed with the protein fold changes between primary tumor and metastasis. Blue and red colors represent up‐and downregulated pathways in primary tumors, respectively, regardless of condition

A total number of 12,657 phosphopeptides were identified across all datasets (Table S3). Despite the variations, the overlap between the total number of phosphopeptides identified across FFPE and FFT conditions was 88.4% (Figure 2A). The normalized phosphopeptide abundances show a strong correlation (Pearson correlation, *r* = 0.89) between FFPE and FFT samples (Figure 2B, Figure S2). More importantly, 2D enrichment annotation analysis^8^ of the phosphoproteome captured similar biological signatures between primary and metastatic tumors, independently of the sample preservation method (Figure 2C). Primary tumors were characterized by the enrichment of pathways related to epidermis development and apoptosis. Metastases were enriched in processes linked to mitochondrial metabolism and microtubule transport.

**FIGURE 2:**
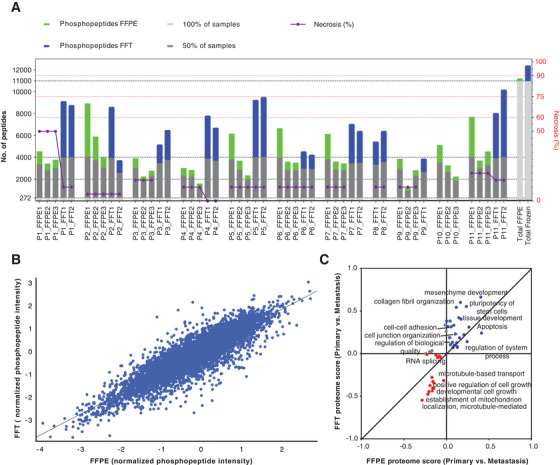
Phosphoproteome analysis of FFPE and FFT tumor samples. (A) Bar graph representing the phosphopeptide coverage by patient and condition FFPE versus FFT. (B) Pearson correlation analysis between the FFPE and FFT samples based on median values of the normalized phosphopeptide abundances (phosphopeptide intensity/total protein intensity) (*r* = 0.89). (C) Comparison between FFPE and FFT conditions based on the enrichment analysis of biological pathways (BP) performed with the phosphopeptides fold changes (log2 ratios) between primary tumor and metastasis (primary/metastasis). Blue and red colors represent BP up‐ and downregulated in primary tumors (respectively), regardless of condition

Since alterations in protein phosphorylation most likely reflect changes in kinase activity levels, we assess the potential of FFPE samples to explore the MM kinome. An integrative bioinformatics analysis was performed to detect and predict the kinases present in the (phospho) proteome datasets. Figure 3A shows the mapping of roughly 70% of the human kinome.^9^ This represents the first large‐scale kinome analysis reported on MM using FFPE samples, supporting the potential of this approach as a tool to discover new pharmaceutical targets.

**FIGURE 3:**
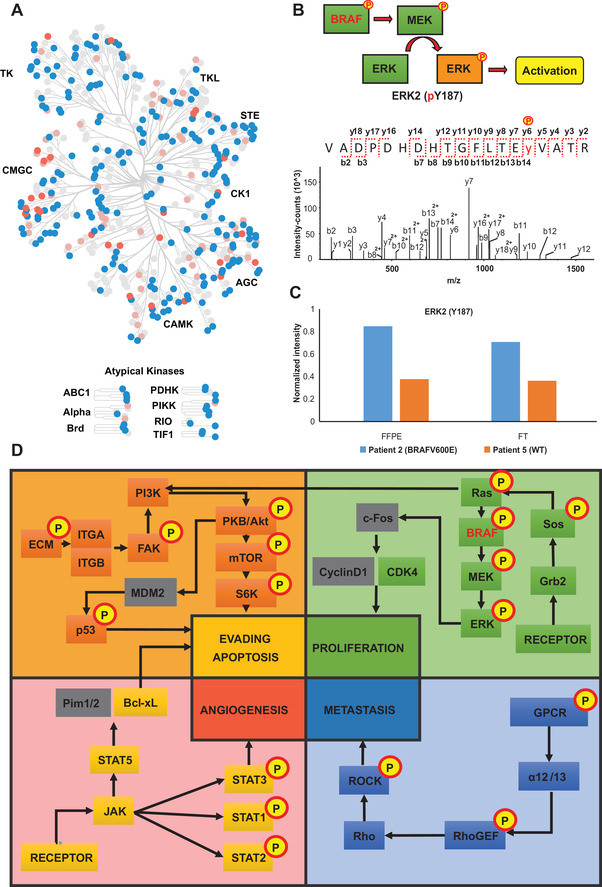
(A) Map of human kinome in malignant melanoma. Blue nodes represent kinases identified in the global proteome dataset. Pink nodes symbolize kinases identified in the phosphoproteome analysis. Coral nodes indicate kinases predicted by phospho‐site analysis. (B) Analysis of BRAF signaling pathway. Assigned MS/MS spectrum corresponding to the phosphopeptide VADPDHDHTGFLTEy(187)VATR of the ERK2 activation site. (C) Quantification of the phosphosite Y187 in a patient with BRAFV600E positive tumor metastasis compared to a patient with WT‐BRAF tumor metastasis for both FFPE and FFT samples. (D) The main cancer signaling pathways mapped by the global proteome and phosphoproteome analysis. The boxes in yellow, orange, green, and blue show proteins experimentally determined. Additionally, the boxes with the yellow circles and “P” display proteins that were found phosphorylated. The grey boxes represent proteins belonging to the indicated biological pathway, but that were not identified in the study

In the era of precision medicine, evaluating patient‐specific molecular profiles of tumors is critical to guide medical decisions, especially to select appropriate pharmacological strategies and to monitor drug resistance. To evaluate the robustness of our analytical pipeline as a tool to monitor patient‐specific molecular events in FFPE versus FFT samples, we investigated the phosphorylation status of crucial oncogenic signaling in MM such as the BRAF pathway (Figure 3B). Multiple BRAF phospho‐sites were unambiguously pinpointed (S365, S446, S729) as well as phosphorylated ERK1 (Y204, T202) and ERK2 (T185, Y187) (Figure 3B). Notably, we detect an increase in the phosphorylation of ERK2 at Y187 in a patient with the BRAFV600E mutation (St.IV) (patient 2), compared to a patient (St.III/c.) without this genetic alteration (patient 5) (Figure 3C). Y187 is required for full activation of ERK2 signaling, and the increase in ERK activity is a common resistance mechanism to long‐term kinase inhibitor therapy.^10^ This demonstrates that our approach allows sensitive monitoring of the differential phosphorylation status of cancer‐related pathways of MM in FFPE samples (Figure 3D), which could add a new dimension to the tumor characterization and personalized treatment strategies. Moreover, multiple relevant oncogenic‐related pathways were covered as well as their phosphorylation status (Figure 3D, Figure S3).

In conclusion, our results provide solid evidence of the suitability of FFPE tissue for high‐quality quantitative proteomics analysis even at the PTM level. Our data encourage the use of FFPE tumor blocks from biobank archives for large‐scale clinical phosphoproteomic studies to uncover kinase signaling associated with MM as well as novel therapeutic targets. The ability to quantify a large number of proteins, together with their modification status in a single analysis, is a valuable tool to compile protein profiles to help in the molecular stratification of MM patients. Incorporating proteomics strategies to screen FFPE tumor blocks in routine clinical settings could help to customize personalized treatment for MM patients.

## CONFLICT OF INTEREST

The authors declare no conflict of interest.

## ETHICS APPROVAL AND CONSENT TO PARTICIPATE

Fresh frozen tumors (FFT) and formalin‐fixed paraffin‐embedded (FFPE) tissues samples were obtained from the Department of Dermatology and Allergology of the University of Szeged, Hungary, under informed consent and a clinical protocol (MEL‐PROTEO‐001). The protocol follows the current EU regulations for clinical testing.

## DATA AVAILABILITY STATEMENT

The data that support the findings of this study are available from the corresponding author upon reasonable request.

## Supporting information

Link the Supporting file 1: Methods

Figure S1 Experimental design and general pipeline for the clinical proteomics and phosphoproteomics workflow implemented in the current study.
Figure S2 Correlation analysis of protein (A) and phosphopeptide (B) intensities quantified for individual patients with paired FFT and FFPE samples.
Figure S3 Main oncogenic pathways mapped by the phosphopeptides detected through the analysis of formalin‐fixed and paraffin‐embedded tissue of Malignant Melanoma samples. Green boxes represent the proteins that participate in the signaling pathway but whose phosphorylated peptides were not detected. The pink boxes represent the proteins whose phosphorylated peptides were identified and quantified in this study

Table S1 Clinical information of the patient cohort

Table S2: Global proteome analysis

Table S3: Phosphoproteome analysis
