# Supplementary material for: A biobanking turning‐point in the use of formalin‐fixed, paraffin tumor blocks to unveil kinase signaling in melanoma
Source: Clin Transl Med. 2021 Aug 4;11(8):e466. doi: 10.1002/ctm2.466 (PMC8335964; doi:10.1002/ctm2.466)
Supplement: Supplementary file 1 — Link the Supporting file 1: Methods [file CTM2-11-e466-s001.docx]

1. METHODS

1.1. Sample selection and histopathological analysis

Primary and metastatic tumors derived from 11 patients diagnosed with MM were collected and preserved as FFPE and FFT. Institutional IRB approved all procedures (Semmelweis University of Szeged, MEL-PROTEO-001001; TUKEB_191-4/2014) protocols with informed patient consent. After surgical removal, the tumor was cut into several pieces. For nine patients, two tumor pieces were preserved as FFT and one as FFPE block. FFT samples were sectioned in the cryostats (LEICA CM1950), collecting ten slices of 10 μm. In the case of the FFPE, we analyzed three tumor layers for proteomic analysis, collecting four sections of 10 µm each. In summary, we processed five samples for these nine-paired patients. According to the preservation technique (FFPE or FFT), the unpaired samples (n=2) were treated according to the same criteria described above, totalizing fifty samples analyzed in this study. In a total of 49 tumor samples were analyzed.

For histological analysis, the sections were collected in a glass and stained with hematoxylin-eosin. All histopathological subtyping has been attributed and aligned by high-level interrogation, according to Melanoma classification provided by the WHO, (https://www.who.int/) introduced at the Paris workshop (26-27 April 2018).

1.2. Deparaffinization of FFPE samples and protein extraction

FFPE samples were incubated in 1 mL EnVision Agilent (dilution 1:50) for 10 min at 97˚C, 500 rpm. After incubation, the samples were centrifuged at 14000 g, 3 min, 4˚C. The supernatant containing paraffin was removed, and the process was repeated four times until all paraffin was cleared out. Deparaffinized samples were resuspended in 500 μL of protein extraction buffer (25mM DTT, 10 % (w/v) SDS in 100mM TEAB pH 8.0), vortexed and incubated at 99 ˚C for 1 hour at 500 rpm.

FFT samples were resuspended in 300 μL of protein extraction buffer (25mM DTT, 10 % (w/v) SDS in 100mM TEAB pH 8.0), vortexed, and incubated at 99 ˚C for 5 min at 500 rpm. After this process, all samples were sonicated (40 cycles, 15s on/off) in the Bioruptor (Diagenode) followed by incubation at 99˚C for 10 min and centrifuged at 20000 g for 20 min at 18˚C. The supernatant was transferred to a new tube and stored at -80 ˚C until further use. An aliquot of the samples was saved for protein determination (660 nm Protein Assay/ Ionic Detergent Compatibility Reagent– ThermoFisher)^11^.

1.3. Protein Digestion, C18 cleaning, and phosphopeptide enrichment

The samples were alkylated with 50 mM IAA for 30 min in the dark at RT. Protein digestion was performed in the S-Trap™ 96-well plate as previously described.^2^ Briefly, 95μL of 50 mM TEAB containing LysC (enzyme: substrate,1:50 ) was added to each sample, and incubated for 2 h, at 37 °C, followed by the addition of 30 µL of digestion buffer (50 mM TEAB) containing Trypsin (enzyme: substrate,1:50), and incubated overnight, at 37 °C. Reaction was stopped by adding 40 µL of 100% formic acid (FA). The peptides were dried in a speed-vac and resuspend in 0.1% trifluoroacetic acid (TFA)/2%acetonitrile (ACN) to perform peptide determination.

One microgram peptide was injected in the LC-MS/MS system for global proteome analysis. For phosphoproteome analysis, the samples were resuspended in 0.1% TFA, and 50 µg of peptides were submitted to an automatic workflow on the AssayMAP Bravo system (Agilent Technologies) for C18 cleaning and Fe(III)-IMAC-based phosphopeptide enrichment as previously described .^3^

1.4. LC-MS/MS analysis and database searching

One microgram of the peptides was injected in two technique replicates in the UltiMate 3000 RSLCnano system (Dionex) coupled to a Q-Exactive HF-X mass spectrometer (Thermo Scientific). A trap-column (Acclaim® PepMapTM 100, 75 μm × 2 cm, nanoViper 2Pk, Thermo scientific) and an analytical column (PepMap RSLC C18, 2 μm, 100 Å, 75 μm x 25 cm Thermo Scientific) were used. The nanoLC was performed in a linear gradient of 160 min, using 0.1% FA (buffer A), and 0.1% FA/ 80% ACN (buffer B) solution in a flow-rate of 300 nL min−1. Mass spectrometer settings, for all the analysis was used in a data-dependent acquisition mode, spray voltage at 1.85 kV and a dynamic exclusion time of 30 s.

For total proteome analysis a chromatographic gradient of 0-3 min 2% of B, 3-115 min 25% of B, 115-125 min 32%B, 125-132 min 45%B, 132-140 90%B 140-145 90%B. The full scan resolution was 120000, AGC 3e6, 50 ms of injection time (IT) was used. For the MS2 analysis the top 20 most intense ions were selected for higher-energy collisional dissociation (HCD) using a resolution of 15000, AGC 1e5, 50 ms of IT and normalized collisional energy (NCE) of 28. The phosphoproteome analysis was performed using a gradient of 0-3 min 4% of B, 3-139 min 30% of B, 139-154 min 45% B, 154-155 min 98% B, 155-160 98% B. Full MS resolution of 120000, AGC 3e6, 50 ms of IT. For the MS2 acquisition, the 15 most intense ions were fragmented and analyzed at a resolution of 60000, AGC 1e5, 120 ms of IT and 25 of NCE.

1.5. Database searching

Mass spectra were searched in the Proteome Discoverer 2.4 (Thermo Scientific) against the UniProt human database (2020/05/26). The global proteome analysis workflow included the Precursor Detector and MSPepSearch nodes. Two spectral libraries (Proteome tools HCD 28 PD and NIST Human Orbitrap HCD) were used for the analysis. In general, mass tolerance for the precursor and fragment were setting as 10 ppm and 0.02 Da, respectively. Maximum 2 missing cleavage sites were accepted for the trypsin digestion.

The pipeline search combines three nodes of the spectrum confidence filter tool. In the first node, methionine oxidation and lysine methylation of peptides, the acetylation, the met-loss, and the met-loss+Acetyl of the protein N-terminal were set as dynamic modifications. Carbamidomethylation of cysteine was set as a static modification. The second node of the spectrum confidence filter also considers as dynamic modifications the methionine oxidation and lysine methylation of peptides, and only the acetylation at protein N-terminal. The cysteine carbamidomethylation of peptides was set up as a static modification. Finally, the last node includes the same dynamic and static modifications as the previous node. In addition, peptide deamidation and carbamylation, and the Gly-pyro-Glu of the peptide N-terminal were set as dynamics modifications. In addition, the Minora Feature Detector and the Feature Mapper node were used during the search.

Phosphoproteome analysis allows a maximum of 3 missing cleavages for the trypsin and a precursor and fragment mass tolerance of 10 ppm and 0.02 Da, respectively. The dynamic modifications were the methionine oxidation and the phosphorylation of S, T, Y at peptide level. The acetylation, met-loss, and met-loss+Acetyl were fixed as dynamic modifications in the protein N-terminal. The carbamidomethylation of the cysteines was included as a static modification in the peptides. The phospho search used the Minora feature detector, the IMP-ptmRS nodes, and the Feature Mapper during the search.

1.6. Bioinformatics analysis

Perseus software (version 1.6.13.0)^4^ was used for the pre-processing of all results. The data was log2 transformed and filtered to keep only proteins with at least three valid values per group (conditions= FFPE or FFT). Global proteome analysis was standardized by cluster normalization strategy. On the other hand, for phosphoproteome data the z-score normalization considering the median of each sample was used. Additionally, the phosphopeptide data was normalized against the total protein abundance to obtain the degree of phosphorylation for the phosphosite. The Pearson correlation analyses were made in the Orange (version 3.25.0).^5^ The 2D pathway enrichment analysis (p= 0.01)^6^ was based on the proteins fold changes using the Perseus software (version 1.6.13.0). The detection of relevant biological pathways in MM cancer was done in the Cytoscape 3.8.2^7^ together with the KEGG Mapper – Search&Color Pathway.^8^ The NetworKIN 3.0 software^9^ was used for the phosphorylation motif analysis. The customizable visualization of the identified and predicted kinase against the human kinome was performed in the CORAL interactive web application.^10^

2. REFERENCE

1. Kuras M, Woldmar N, Kim Y, et al. Proteomic Workflows for High-Quality Quantitative Proteome and Post-Translational Modification Analysis of Clinically Relevant Samples from Formalin-Fixed Paraffin-Embedded Archives. *J Proteome Res*. 2021;20(1):1027-1039. doi:10.1021/acs.jproteome.0c00850

2. PROTIFI. S-Trap^TM^ 96-well plate digestion protocol. https://files.protifi.com/protocols/s-trap-96-well-plate-long-1-4.pdf.

3. Murillo JR, Kuras M, Rezeli M, Milliotis T, Betancourt L, Marko-Varga G. Automated phosphopeptide enrichment from minute quantities of frozen malignant melanoma tissue. *PLoS One*. 2018;13(12):1-15. doi:10.1371/journal.pone.0208562

4. Tyanova S, Temu T, Sinitcyn P, et al. The Perseus computational platform for comprehensive analysis of (prote)omics data. *Nat Methods*. 2016;13(9):731-740. doi:10.1038/nmeth.3901

5. Demsar J, Curk T, Erjavec A, Gorup C, Hocevar T, Milutinovic M, Mozina M, Polajnar M, Toplak M, Staric A, Stajdohar M, Umek L, Zagar L, Zbontar J, Zitnik M ZB. Orange: Data Mining Toolbox in Python. *J Mach Learn Res*. 2013;14(Aug): 2.

6. Cox J, Mann M. 1D and 2D annotation enrichment: a statistical method integrating quantitative proteomics with complementary high-throughput data. *BMC Bioinformatics*. 2012;13 Suppl 1(Suppl 16):S12. doi:10.1186/1471-2105-13-S16-S12

7. Paul Shannon 1, Andrew Markiel 1, Owen Ozier, 2 Nitin S. Baliga, 1 Jonathan T. Wang, 2 Daniel Ramage 2, et al. Cytoscape: A Software Environment for Integrated Models. *Genome Res*. 1971;13(22):426. doi:10.1101/gr.1239303.metabolite

8. Ogata H, Goto S, Sato K, Fujibuchi W, Bono H, Kanehisa M. KEGG: Kyoto encyclopedia of genes and genomes. *Nucleic Acids Res*. 1999;27(1):29-34. doi:10.1093/nar/27.1.29

9. Linding R, Jensen LJ, Pasculescu A, et al. NetworKIN: A resource for exploring cellular phosphorylation networks. *Nucleic Acids Res*. 2008;36(SUPPL. 1):695-699. doi:10.1093/nar/gkm902

10. Metz KS, Deoudes EM, Berginski ME, et al. Coral: Clear and Customizable Visualization of Human Kinome Data. *Cell Syst*. 2018;7(3):347-350.e1. doi:10.1016/j.cels.2018.07.001
