# Supplementary material for: A biobanking turning‐point in the use of formalin‐fixed, paraffin tumor blocks to unveil kinase signaling in melanoma
Source: Clin Transl Med. 2021 Aug 4;11(8):e466. doi: 10.1002/ctm2.466 (PMC8335964; doi:10.1002/ctm2.466)
Supplement: Supplementary file 2 — Figure S1 Experimental design and general pipeline for the clinical proteomics and phosphoproteomics workflow implemented in the current study. Figure S2 Correlation analysis of protein (A) and phosphopeptide (B) intensities quantified for individual patients with paired FFT and FFPE samples. Figure S3 Main oncogenic pathways mapped by the phosphopeptides detected through the analysis of formalin‐fixed and paraffin‐embedded tissue of Malignant Melanoma samples. Green boxes represent the proteins that participate in the signaling pathway but whose phosphorylated peptides were not detected. The pink boxes represent the proteins whose phosphorylated peptides were identified and quantified in this study [file CTM2-11-e466-s004.pdf]

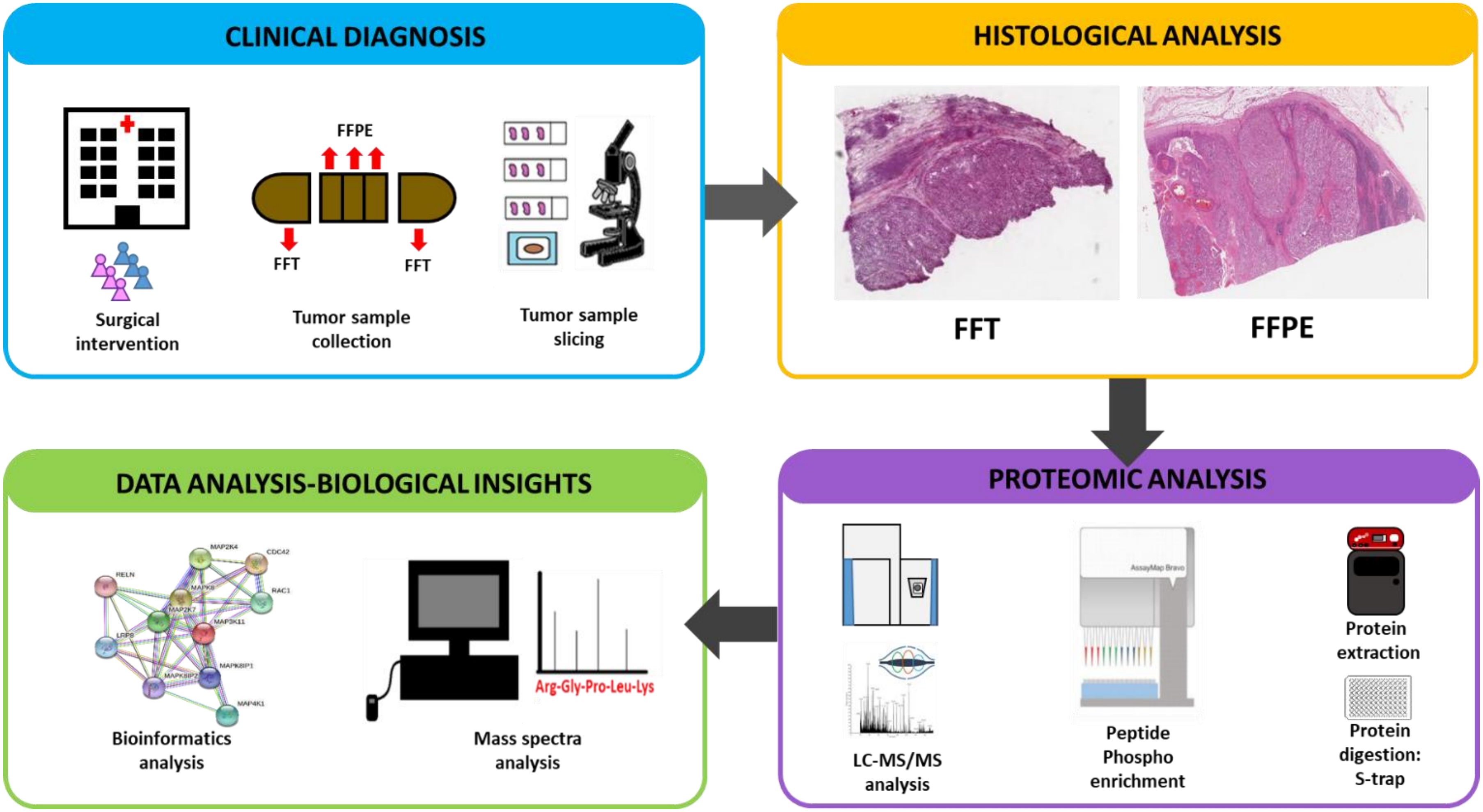

Supporting Figure 1. Experimental design and general pipeline for the clinical proteomics and phosphoproteomics workflow implemented in the current study.

A

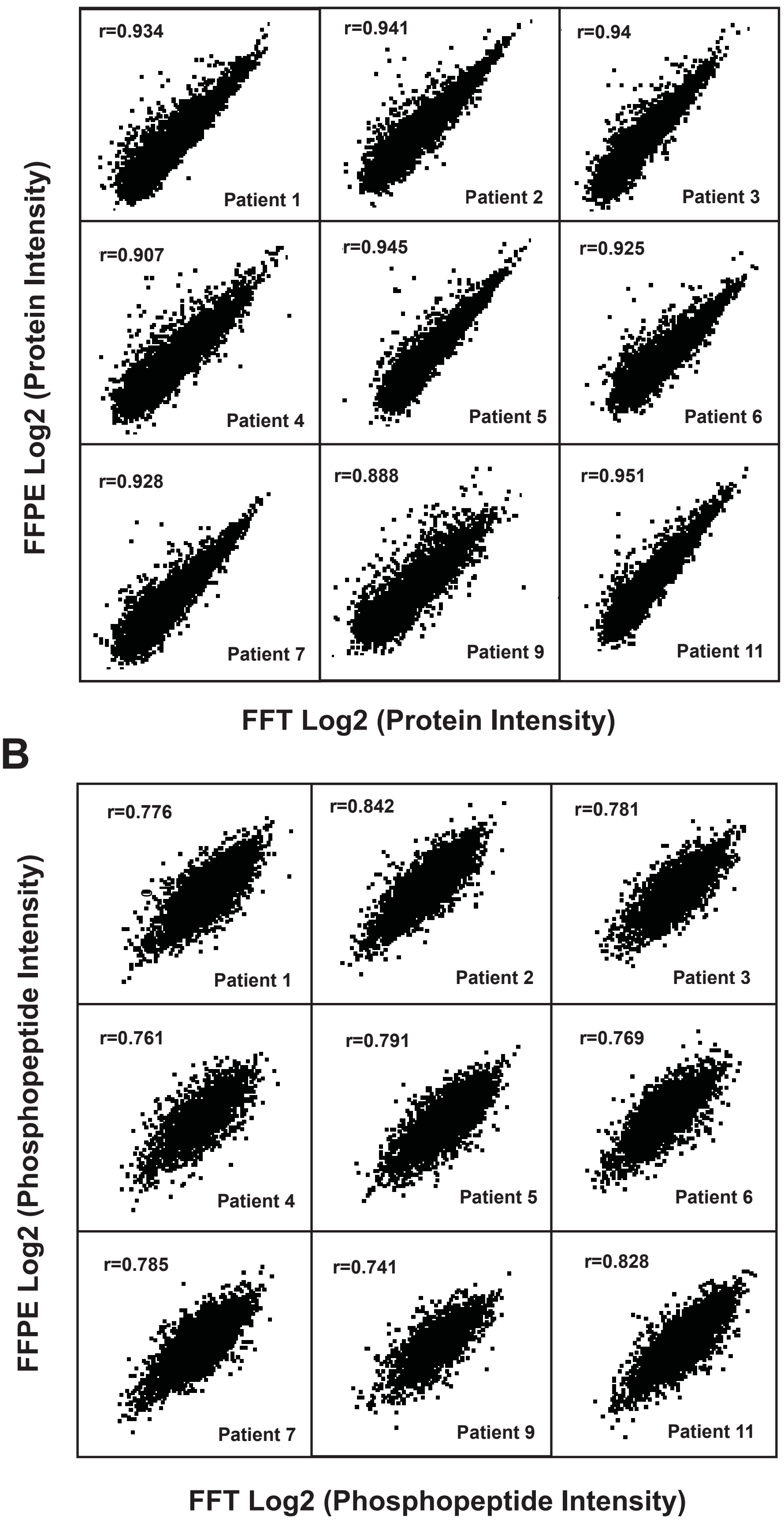

Supporting Figure 2. Correlation analysis of protein **(A)** and phosphopeptide **(B)** intensities quantified for individual patients with paired FFT and FFPE samples.

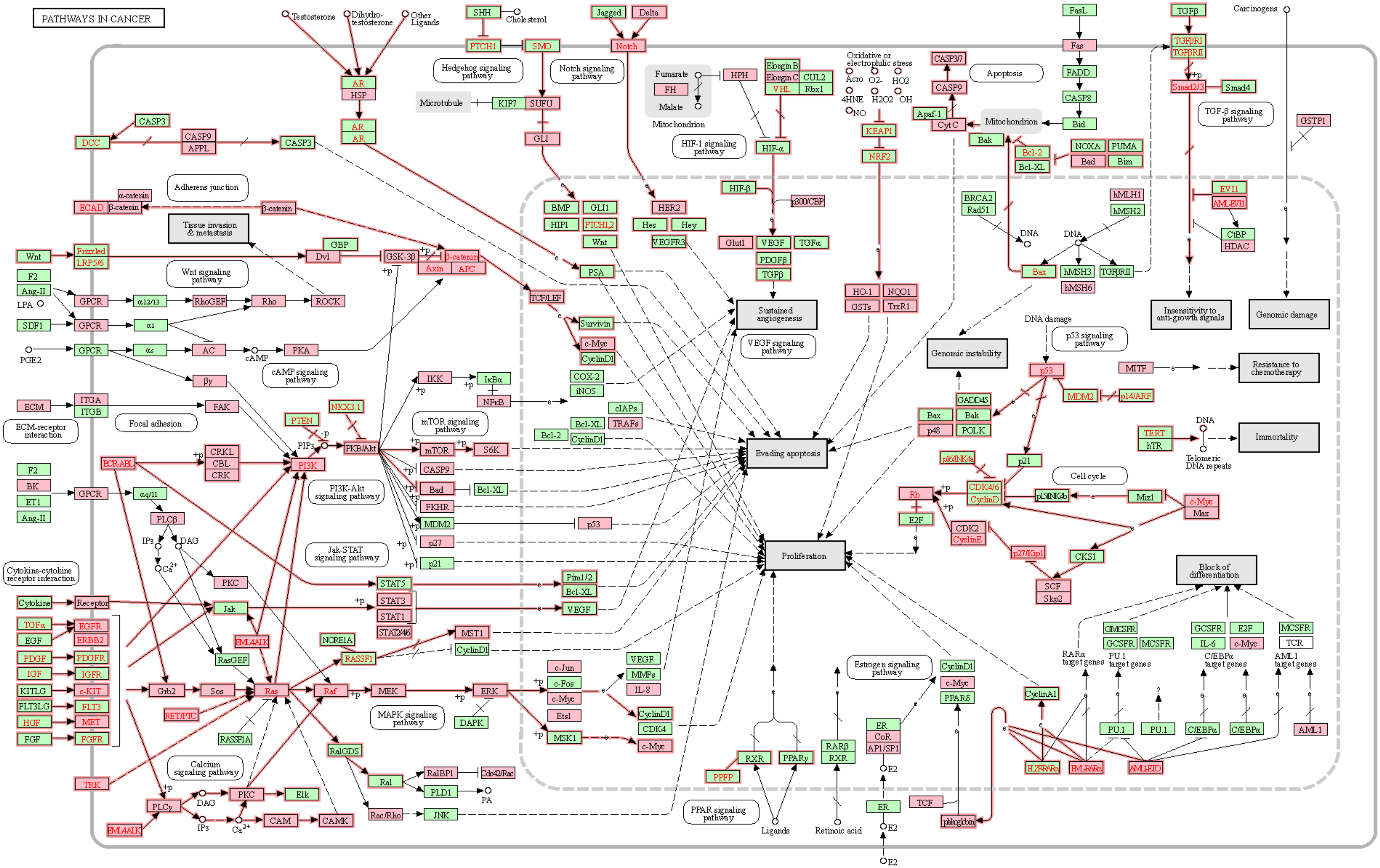

Supporting Figure 3. Main oncogenic pathways mapped by the phosphopeptides detected through the analysis of Formalin-fixed and paraffin-embedded tissue of Malignant Melanoma samples. Green boxes represent the proteins that participate in the signaling pathway but whose phosphorylated peptides were not detected. The pink boxes represent the proteins whose phosphorylated peptides were identified and quantified in this study.
