# Supplementary material for: A biobanking turning‐point in the use of formalin‐fixed, paraffin tumor blocks to unveil kinase signaling in melanoma
Source: Clin Transl Med. 2021 Aug 4;11(8):e466. doi: 10.1002/ctm2.466 (PMC8335964; doi:10.1002/ctm2.466)
Supplement: Supplementary file 3 — Table S1 Clinical information of the patient cohort [file CTM2-11-e466-s003.pdf]

Table S1. Clinical information of the patient cohort

Patient			Collected sample				Clinical data				
							Primary tumor		Clinical stage	BRAF mutational state	
ID	Gender	Age at diagnosis (ys)	FFT	FFPE	Type	Location	Type	Pathological stage		WT	V600E
Patient_1	f	61	y	y	Lymph node metastasis	Inguinal region	SSM	pT1b	III	0	1
Patient_2	f	61	y	y	Cutaneous metastasis	Right breast	SSM vertical growth (not removed since of advanced disease)	pTx	IV	0	1
Patient_3	f	60	y	y	Lymph node metastasis	Axillary region	Melanoma with complete regression	pTx	III	0	1
Patient_4	m	65	y	y	Primary tumor	Lower limb	Malignant blue naevus	pT4a	III	0	1
Patient_5	f	81	y	y	Lymph node metastasis	Inguinal region	ALM vertical growth	pT3b	III	1	0
Patient_6	f	96	y	y	Primary tumor	Head and neck region	LMM vertical growth	pT4b	II	1	0
Patient_7	m	59	y	y	Lymph node metastasis	Axillary region	SSM	pT2a	IV	0	1
Patient_8	m	70	y	n	Primary tumor	Trunk	SSM vertical growth (not removed since of advanced disease)	pTx	IV	0	1
Patient_9	m	54	y	y	Primary tumor	Lower limb	ALM vertical growth	pT4b	IV	1	0
Patient_10	m	74	y	y	Primary tumor	Trunk	NM	pT4b	IV	1	0
Patient_11	m	55	n	y	Primary tumor	Lower limb	NM	pT4b	III	0	1
